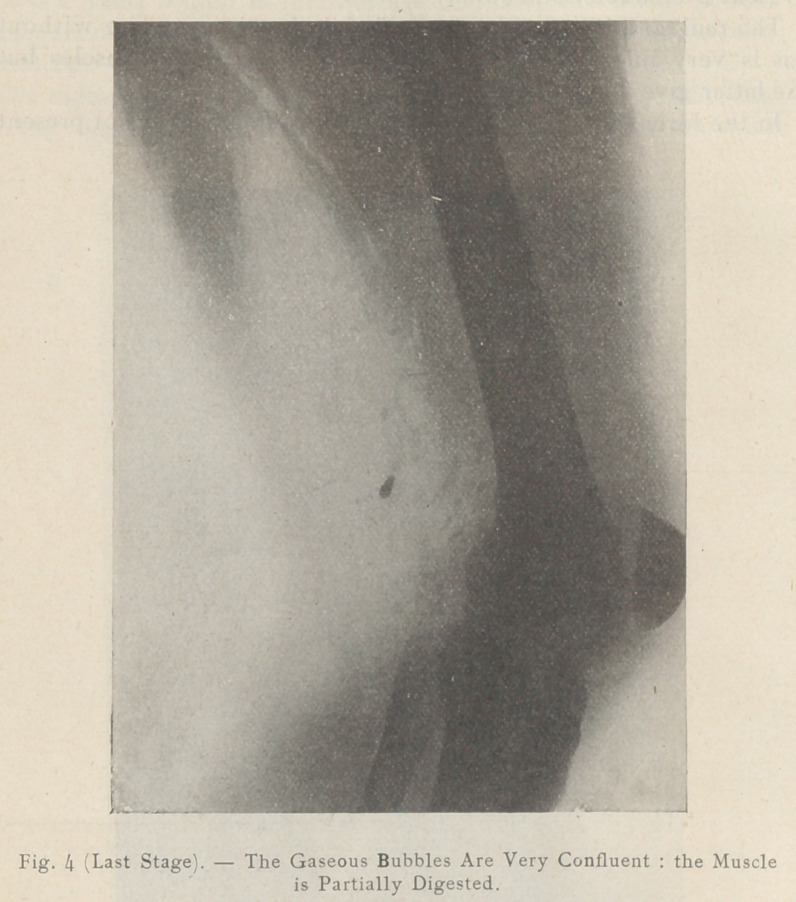# Radiographic Aspects of Gangrenous Infections of War Wounds and of Gas Gangrene in Particular

**Published:** 1917-12

**Authors:** 


**Radiographic aspects of Gangrenous Infections of War Wounds and of Gas Gangrene in Particular.** By MM. G. LARDENNOIS and PECH. Trans. from the *Journal de Radiologie et d'Electrologie*, May-June, 1917.

By means of suitably chosen rays it is possible to obtain radiographic plates of muscles. Following the example of the

Fig. 1 (1st Stage). — Progressive Destruction of the Leg Muscle along an Infected Tract.

Americans and of Ledoux-Lebard in France, we have applied this method to the study of gangrenous infections of war wounds.

A plate taken of a sound limb shows a gray image of fleshy masses, almost homogeneous.

A plate taken of a swollen limb infected by the usual pyogenic organisms gives an aspect only slightly different. The image shows a limb which is increased in volume, the shade is still homogeneous but a little uncertain, light lines mark the muscular interstices.

The radiographic aspect of a gangrenous limb is very different. At the beginning during the stage of malign and muscular

Fig. 2 (2nd Stage). — A Fascicular Group of Muscles of the Leg in Process of Digestion.

tumefaction it is easy to follow the progress of destruction of the muscles around the infected track, where a light zone with irregular outline becomes visible.

At the second stage the destruction has progressed and the gangrene becomes diffuse. One can then see on the plate spots and striae delineating the muscular bundles in course of digestion.

At a later stage the clear spaces are enlarged. The muscular compartments appear occupied by bubbles of irregular outline and present a characteristic cloudy aspect.

The radiographic aspect of putrified ischemic gangrene without gas is very different. Wide light spaces separate the muscles but the latter give a normal shadow.

In the form of pure malignant edema, the image does not present

Fig. 3 (Last Stage). — The Muscular Fibers of the Thigh Invaded by Bubbles of Irregular Outline.

any of the characteristic aspects that we have just described.

To a competent radiographer, the fluorescope gives the same findings.

The information furnished by a radiographic examination of gas gangrene is not merely of interest for the study of the method of destruction by the anaerobes and the localization of the process in the muscles. It can also be very useful for the diagnosis of a point of gangrene and even more for the evaluation of its extent. In

Fig. 4 (Last Stage). — The Gaseous Bubbles Are Very Confluent : the Muscle is Partially Digested.

certain cases this information has rendered valuable service and has permitted us to institute a rational treatment.

(The plates are reprinted be courtesy of the *Journal de Radiologie et d'Electrologie.*)

## Figures and Tables

**Fig. 1 (1st Stage). f1:**
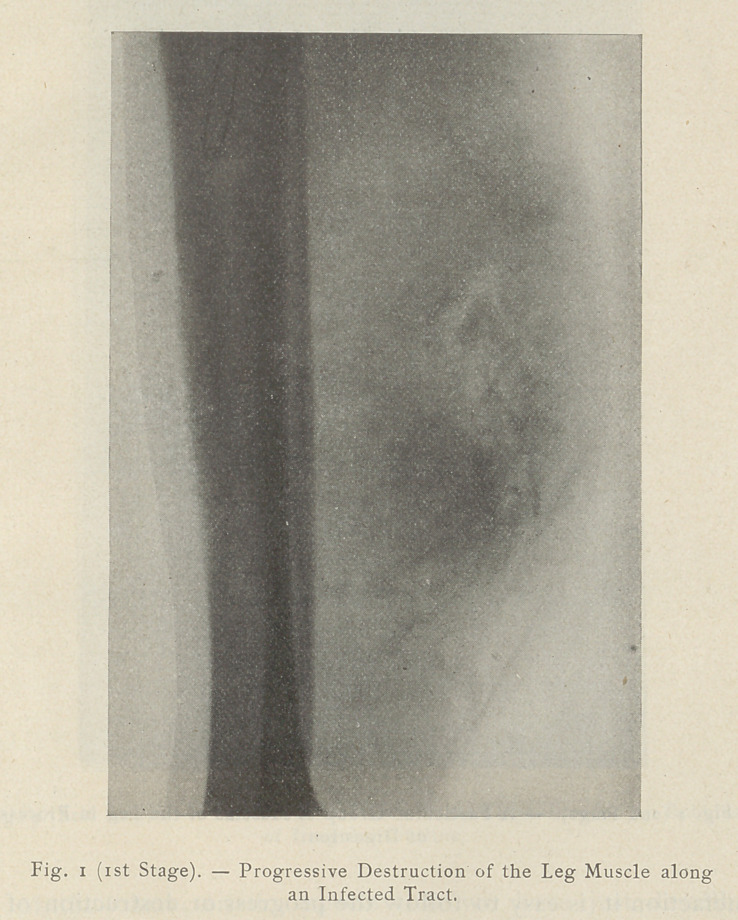


**Fig. 2 (2nd Stage). f2:**
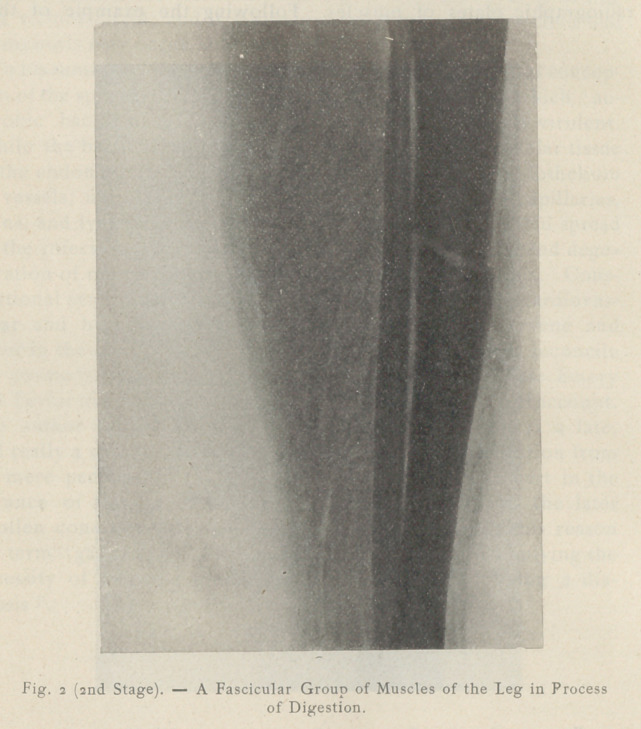


**Fig. 3 (Last Stage). f3:**
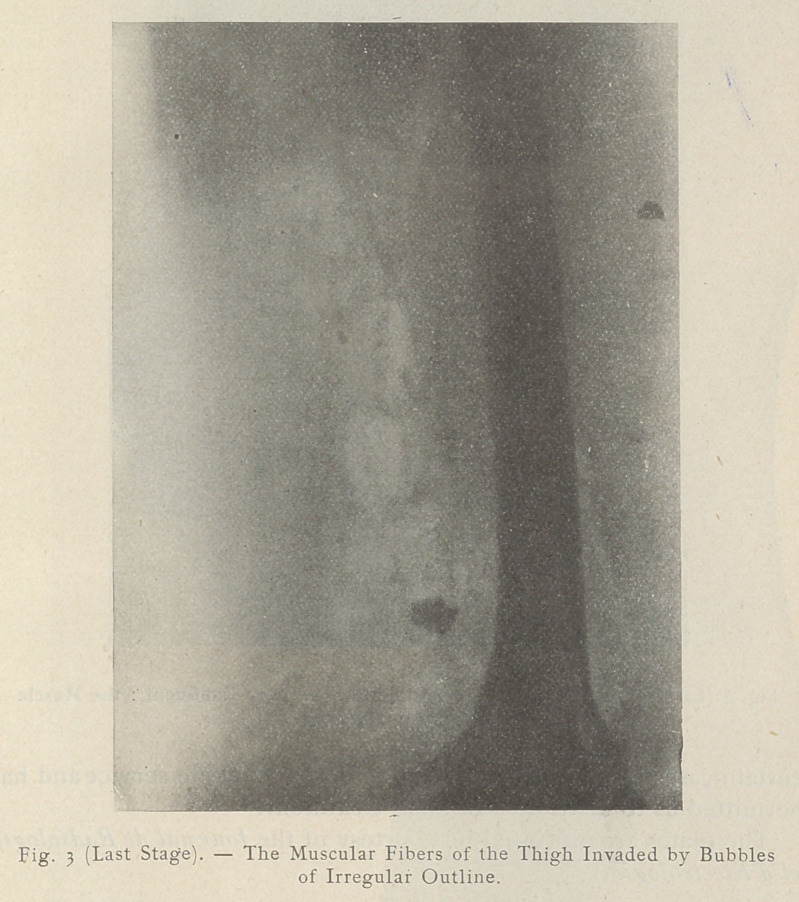


**Fig. 4 (Last Stage). f4:**